# The Digital Shift in the Post-COVID Era: Evaluating the Impact of Web-Enabled Education on Cognitive, Affective, and Psychomotor Skill Enhancement in Gen ‘Z’ Nursing Students- A Systematic Review with meta-analysis.

**DOI:** 10.17533/udea.iee.v43n2e09

**Published:** 2025-07-25

**Authors:** Joyce Robert Mathivanan, Seeta Devi

**Affiliations:** 1 Nurse, Tutor, M.Sc. Email: mathivananjoyce@gmail.com. Corresponding Author. https://orcid.org/0009-0009-7433-4170 Symbiosis International University India mathivananjoyce@gmail.com; 2 Nurse, Ph.D. Professor & HoD. Email: drseetadevi1981@gmail.com. https://orcid.org/0000-0002-6220-7264 Symbiosis International University India drseetadevi1981@gmail.com; 3 Symbiosis College of Nursing, Symbiosis International (Deemed University), Pune, Maharashtra, India Symbiosis International University Symbiosis College of Nursing Symbiosis International (Deemed University) Pune Maharashtra India

**Keywords:** educational technology, internet-based intervention, computer-assisted instruction, students, nursing, education, nursing, clinical decision-making., tecnología educativa, intervención basada en la internet, instrucción por computador, estudiantes de enfermería, educación en enfermería, toma de decisiones clínicas., tecnologia educacional, intervenção baseada em internet, instrução por computador, estudantes de enfermagem, educação em enfermagem, tomada de decisão clínica.

## Abstract

**Objective.:**

This study evaluates the impact of digital educational technologies on nursing students' academic achievement and learning outcomes.

**Methods.:**

A literature review was conducted to identify peer-reviewed articles published in English between 2020 and June 2024 in databases such as Web of Science, Science Direct, EBSCO, NLM/NIH/PMC and Scopus. This review compares the efficacy of digital educational interventions against control groups using Cochrane Collaboration's risk of bias (RoB) and Standardized Mean Difference (SMD) for outcome measurement, with results analyzed using RevMan Web.

**Results.:**

This study analyzed fourteen randomized controlled trials involving 1611 participants. The meta-analysis found that digital educational technologies enhanced nursing students' cognitive skills (SMD=0.45; *p*<0.001), critical thinking and clinical decision-making skills (SMD=0.88; *p*<0.001), attitudes (SMD=0.94; *p*<0.001), and clinical skills (SMD=1.09; *p*<0.001) when compared to conventional instructional methods. Nevertheless, there was no statistically significant improvement in the problem-solving ability (SMD=1.00, *p*=0.07).

**Conclusion.:**

Recent advances in digital technology provide a spectacular opportunity to improve healthcare practices for nurses and nursing students. Their integration can potentially increase educational and professional skills, quality of life, and patient satisfaction.

## Resumen

**Objetivo.:**

Evaluar el impacto de las tecnologías educativas digitales en el rendimiento académico y los resultados de aprendizaje de los estudiantes de enfermería.

**Métodos.:**

Se realizó una búsqueda bibliográfica para identificar artículos revisados por pares publicados en inglés entre 2020 y junio de 2024 en bases de datos Web of Science, Science Direct, EBSCO, NLM/NIH/PMC y Scopus. Esta revisión comparó la eficacia de las intervenciones educativas digitales frente a grupos de control, se evaluó el riesgo de sesgo (RoB) de la Colaboración Cochrane y la Diferencia de medias estandarizada (DME) para la medición de resultados.

**Resultados.:**

Se analizaron catorce ensayos controlados aleatorios con 1611 participantes. El metaanálisis mostró que las tecnologías educativas digitales mejoraron las habilidades cognitivas de los estudiantes de enfermería (DME=0.45; *p*<0001), las habilidades de pensamiento crítico y toma de decisiones clínicas (DME=0.88; *p*<0001), las actitudes (DME=0.94; *p*<0001) y las habilidades clínicas (DME=1.09; *p*<0001) en comparación con los métodos de instrucción convencionales. Sin embargo, no hubo una mejoría estadísticamente significativa en la capacidad de resolución de problemas (DME=1.00, *p*=0.07).

**Conclusión.:**

Los recientes avances en tecnología digital mejoraron significativamente las prácticas asistenciales de las estudiantes de enfermería y su integración puede aumentar potencialmente las competencias educativas y profesionales, la calidad de vida y la satisfacción de los pacientes.

## Resumo

**Objetivo.:**

Avaliar o impacto das tecnologias educacionais digitais no desempenho acadêmico e nos resultados de aprendizagem de estudantes de enfermagem.

**Métodos.:**

Foi realizada uma revisão bibliográfica para identificar artigos revisados por pares publicados em inglês entre 2020 e junho de 2024 em bases de dados como a *Web of Science*. *Science*, *Science Direct*, EBSCO, NLM/NIH/PMC e *Scopus*. Esta revisão compara a eficácia das intervenções educacionais digitais versus grupos de controle usando o risco de viés (RoB) e a diferença média padronizada (SMD) da *Cochrane Collaboration* para medição de resultados, com resultados analisados usando *RevMan Web*.

**Resultados.:**

Quatorze ensaios clínicos randomizados com 1611 participantes foram analisados. A meta-análise mostrou que as tecnologias educacionais digitais melhoraram as habilidades cognitivas dos estudantes de enfermagem (SMD = 0.45; *p*<0.001), o pensamento crítico e as habilidades de tomada de decisão clínica (SMD = 0.88; *p*< 0.001), as atitudes (SMD = 0.94; *p*<0.001) e as habilidades clínicas (SMD = 1.09; *p*<0.001) em comparação com os métodos instrucionais convencionais. No entanto, não houve melhora estatisticamente significativa nas habilidades de resolução de problemas (SMD = 1,00, *p* = 0,07).

**Conclusão:**

Os avanços recentes na tecnologia digital melhoraram significativamente as práticas de saúde dos estudantes de enfermagem, e sua integração pode potencialmente melhorar as competências educacionais e profissionais, a qualidade de vida e a satisfação do paciente.

## Introduction

In nursing education worldwide, digital learning platforms, whether used independently or in conjunction with traditional teaching methods, have become integral to developing practical competencies among undergraduate students.^1^ With the rapid pace of technological advancement, educational institutions have increasingly embraced online instructional tools, recognizing their potential to transform learning environments.^2^ While digital learning platforms have been utilized in educational sciences since the 1980s, their application in nursing education remains relatively recent.^3^ The primary goal of nursing programs is to prepare students to graduate as highly competent and confident professionals. Nursing education relies on both theoretical knowledge and practical skills, such as in-person, hands-on training, where recall ability is often achieved through visual presentations, note-taking, and interactive learning techniques.^4^ Guven *et al.* suggested that incorporating e-learning alongside traditional face-to-face instruction can significantly enhance the development of clinical skills.^5^

Before the COVID-19 pandemic, nursing students were ready to embrace digital learning and acknowledged its value in education. However, persistent challenges, including insufficient technical support and the stress associated with technology use, posed barriers to its seamless adoption.^6^The pandemic, however, necessitated an extraordinary and immediate shift to digital-based education, particularly in disciplines, including the nursing profession, that heavily demands hands-on training. This abrupt transition emphasized the critical need for nursing students to acquire clinical competence through innovative teaching and learning technologies, as traditional in-person experiences were no longer feasible.^7^ Globally, social distancing mandates disrupted higher education, compelling institutions to pivot to distance learning almost overnight.^8^ The focus shifted to virtual clinical experiences, emphasizing participative learning and leveraging digital tools to simulate real-world scenarios.^9^ Niigata University in Japan, for instance, faced significant obstacles in securing clinical training placements for Basic Nursing Practicum II during the fiscal years 2020 and 2021 due to restricted hospital access. In response, the university adopted a hybrid high-fidelity simulation model, integrating on-campus, face-to-face sessions with remote online components. This innovative approach maintained the integrity of nursing education by blending time-lapse unfolding case studies with interactive virtual experiences.^10^

E-learning has become a valuable resource for overcoming the limitations of conventional education. It enables nursing students to take an active and accountable role in their learning journey while offering cost-effective, accessible, and lifelong learning opportunities.^11^ Furthermore, online education supports the development of both theoretical knowledge and practical clinical skills essential for nurses to excel in their practice.^12^ Traditional training methods often face challenges due to the demands of clinical practice, including limited access to diverse and flexible learning environments.^13^ Digital educational solutions address these gaps by providing accessible, time-efficient, and scenario-rich learning experiences. Scenario-based instruction combined with reflective feedback strengthens cognitive integration and helps bridge the gap between academic theory and clinical application, preparing student nurses for the dynamic healthcare landscape.^14^

While numerous studies have highlighted the benefits of web-based learning in nursing education, outcomes vary depending on the intervention and assessment criteria. This study aims to evaluate the impact of digital-based educational technology on nursing students’ knowledge acquisition, critical thinking, decision-making, problem-solving abilities, attitude and clinical skill enhancement. To achieve this, a meta-analysis of available randomized controlled trials (RCTs) was conducted. Despite the limited number of RCTs on e-learning in nursing curricula, this analysis provides a comprehensive evaluation of digital-based educational approaches, from online pedagogies to high-fidelity virtual reality simulators, compared to traditional teaching methods. The findings contribute to the ongoing discussion on the integration of digital-based education into modern nursing curricula to enhance student learning and clinical preparedness.

## Methods

Meta-analysis, an effective quantitative research approach, was used to carefully synthesis previous study findings and draw comprehensive conclusions. This study strictly adhered to the Preferred Reporting Items for methodical Reviews and Meta-Analyses (PRISMA) standards^15^ to ensure a clear and methodical approach to reporting. The study topic was carefully crafted utilizing the PICOT framework, with nursing students as the population, web-based learning as the intervention, and traditional learning methods as the comparator. The results of interest were knowledge, critical thinking, decision-making, problem-solving abilities, attitude and clinical skill enhancement with research published after COVID defining the temporal scope. This rigorous methodology provided a precise and focused framework that allowed for a thorough assessment of the influence of digital-based learning on nursing education across multiple crucial domains.

*Search strategy.* An extensive electronic search was carried out across several academic databases, including Scopus, Science Direct, Web of Science, EBSCO, and NLM/NIH/PMC. The review included English-language papers published after COVID. To ensure a thorough and relevant search, the following MeSH terms were employed: “Nursing Students”; “Web-Based Learning”; “Traditional Learning Methods”; “Knowledge”; “Performance Confidence”; “Critical Thinking”; “Decision-Making”; “Problem-Solving Ability”; “Internet -Based Intervention”; Clinical Competence”; “Randomized Control Trials.” Ongoing clinical trials listed on ClinicalTrials.gov and the International Clinical Trial Registry Platform were also reviewed. Furthermore, references from this study and existing systematic reviews were examined to identify any missing studies.

*Criteria for Inclusion and Study Selection.* The process of choosing papers to evaluate the impact of web-based learning in nursing education followed predefined inclusion criteria. The following standards were used to select the studies for inclusion: 1) Nursing students enrolled in colleges, utilizing various web-based learning methods as part of their teaching and learning strategies. 2) Full-text randomized controlled trials (RCTs) published in English between the post-COVID period and June 2024. 3) All randomized controlled trials available up to the search date. 4) Online teaching and learning methods, including internet-based teaching platforms such as Virtual Reality Simulation, Virtual Reality (Video; Online & game-based phone application), Virtual Learning, Simulation (Video-based; Zoom learning), Web-based game, Online Learning, AI-Powered Doctor, Web‐based (Mobile training; Learning). 5) Comparative methods involving traditional teaching approaches, such as theoretical lecture and laboratory teaching, Questionnaire/training booklet/structured guidelines, Self-directed learning using e-books, Offline Low-fidelity simulation and Human-controlled Avatar. The selected studies were required to report pre-test and post-test outcomes, indicating positive, negative, or neutral effects resulting from the use of web-based learning approaches. This systematic and comprehensive approach ensured an integrative assessment of the impact of web-based learning methods on nursing education across diverse modalities and outcome measures.

*Exclusion Criteria.* Studies were excluded if they lacked a clearly stated research question, were limited to case studies, or were presented as conference abstracts or editorial letters.

*Collection of data.* The articles found using the search approach were imported into the Zotero referencing system for management, where duplicate records were carefully removed. Two reviewers separately screened titles, abstracts, and complete texts using the defined inclusion and exclusion criteria. For instances where full texts were inaccessible or clarifications were needed, the authors contacted researchers via email and academic platforms like Google Scholar and Research Gate. Any differences that developed during the evaluation process were resolved through detailed discussions. If no consensus could be achieved, the case was referred to a third reviewer, who made the final decision. All studies excluded from the review were recorded, along with the reasons for their exclusion.

*Assessment for risk bias.* The included studies were evaluated for bias using the Cochrane Collaboration Risk of Bias Tool, version 2,^16^ and their quality was assessed using the Joanna Briggs Institute (JBI) Critical Appraisal Tool.^17^ Two reviewers separately completed these evaluations. When the reviewers differed, a third reviewer was engaged to reconcile the differences before finalizing the summary and grade of the overall risk of bias.

*Quality Assessment.*Figures 2 and 3 show a visual depiction of the RoB and an evaluation of methodological quality for fourteen RCTs. Among these, three studies ^18^^-^^20^ presented an unclear risk related to participant blinding, allocation concealment, and missing data (Figures 2 and 3) while eleven studies^21^^-^^31^ demonstrated a low risk of bias. Furthermore, regarding methodological quality, five studies ^18^^,^^19^^,^^26^^,^^27^^,^^29^ were identified as having moderate quality, while the remaining nine studies ^20^^-^^25^^,^^28^^,^^30^^,^^31^ were rated as high quality.

*Data Analysis.* The web-based version of Cochrane's Review Manager software (RevMan) was used to analyze the data. A 95% CI random-effects model was used to demonstrate the meta-analysis results as standardized mean differences. The study's heterogeneity was assessed using the I² statistic. However, the small number of studies included the conduct of additional subgroup analysis.

## Results

### Population Characteristics and Study Selection

**Figure 1:**
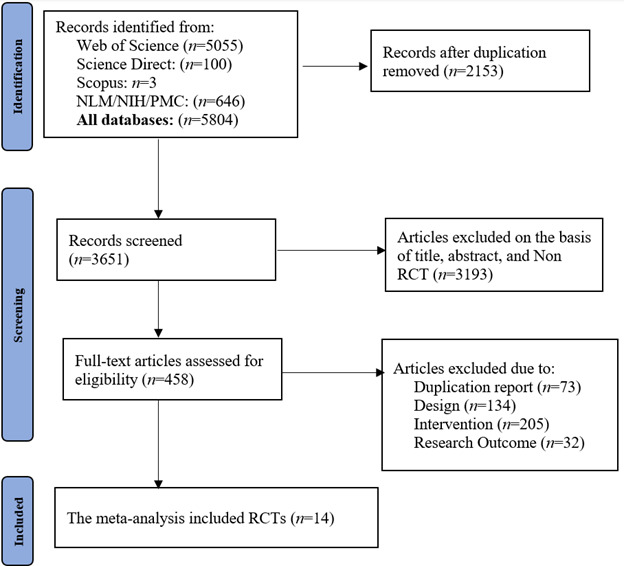
PRISMA Flow diagram. Key Features of Included RCTs Comparing Digital and Traditional Educational Methods

Our comprehensive literature search yielded a total of 5,804 studies from various databases: Web of Science (*n*=5055), Science Direct (*n*=100), Scopus (*n*=3), NLM/NIH/PMC repositories (*n*=646). After eliminating duplicate entries, the pool was reduced to 458 unique articles. These were then meticulously evaluated against our predefined inclusion criteria. Ultimately, only 14 studies^18^^-^^31^ fully satisfied these rigorous standards and were consequently incorporated into our systematic review. As shown in Figure 1. 

This research encompasses the main characteristics of 14 studies evaluating educational interventions that employed both digital and traditional teaching methodologies for nursing students. Comprehensive details are outlined in Table 1. Regarding the design framework, sample size, exposure, and outcome measures, all included studies adhered to a randomized controlled trial (RCT) design. The total sample size was 1,611 participants, with 868 nursing students assigned to the experiment group and 793 to the control group.

**Table 1:** Outlines the features of the 14 studies chosen for inclusion in the systematic review

Reference	Design	Sample size	Exposure	Outcome Measures				Quality Assessment tool (JBI)		
				Knowledge		Critical thinking & clinical decision making			Problem solving ability		Attitude		Clinical Skill		
				I	II	I	II	I	II	I	II	I	II	
21	RCT	87 (E:42, C:45)	E: Video-Based Simulation Training on Perioperative Process	20.38 ± 7.41	22.14 ± 9.84	169.60± 11.82	171.86± 10.22	-	-	-	-	-	-	High 11/13
			C: Theoretical Lecture and laboratory teaching on post-operative patient care	19.96 ± 8.35	18.36± 8.35	168.26± 9.53	166.23± 12.73	-	-	-	-	-	-	
22	RCT	72 (E:36, C:36)	E: Blended learning approach, integrating virtual simulation with interactive operation performance online for CPR Skills	-	-	277.75 ± 25.47	286.64 ± 26.678	-	-	-	-	-	-	High 12/13
			C: Low-fidelity simulation offline for teaching CPR skills	-	-	267.22 ± 22.221	274.11 ± 29.399	-	-	-	-	-	-	
23	RCT	44 (E:22, C:22)	E: two‐week mobile web‐based training programme covering key topics in patient safety management	18.68± 5.46	18.55± 5.23	-	-	-	-	3.38± 0.36	4.01± 0.39	3.26± 0.40	3.93± 0.4	High 11/13
			C: Training booklet.	10.91± 5.48	12.36± 4.85	-	-	-	-	3.68± 0.32	3.70± 0.39	3.47± 0.57	3.5± 0.57	
18	RCT	328 (E:226, C:102)	E: Simulation-based Zoom learning (SBZL)	-	-	-	79.69 ± 3.96	3.95 ± 0.39	3.99 ± 0.41	-	-	-	-	Moderate 9/13
			C: Traditional method	-	-	-	77.69 ± 6.31		4 ± 0.45	-	-	-	-	
24	RCT	228 (E:128, C:100)	E: Virtual learning strategy	0.79 ± 2.15	5.12 ± 2.46	-	-	-	-	-	-	-	-	High 12/13
			C: Active teaching methods	0.74 ± 2.25	4.75 ± 2.76	-	-	-	-	-	-	-	-	
25	RCT	57 (E:27, C:30)	E: 360º VR video C: face-to-face demonstration	5 (*p*=0.87)	15 (*p*=0.952)	-	-	-	-	-	-	-	30 (p=0.273)	High 11/13
5 (*p*=0.87)	16 (*p*=0.952)	-	-	-	-	-	-	-	32 (p=0.273)
26	RCT	50 (E:25, C:25)	E: VR-based simulation training on geriatric oral health care	-	8.92±0.70	-	-	-	-	-	28.92±2.6	35.28±6.15	42.05±4.19	Moderate 10/13
			C: Questionnaire	-	7.28±1.34	-	-	-	-	-	27.92±3.75	34.08±4.86	35.84±5.61	
27	RCT	64 (E:32, C:32)	E: AI-powered doctor (AI-powered group)	6.91±1.63	9.06±1.78	-	-	-	-	-	-	-	13.63±4.23	Moderate 10/13
			C: virtual reality simulation (human-controlled group)	7.03±2.25	7.75±2.08	-	-	-	-	-	-	-	12.75±3.85	
28	RCT	299 (E:101, C:98)	E: Online problem-based learning intervention on self-based learning	-	-	-	163.15±17.94	-	-	-	-	-	82.97±11.5	High 13/13
			C: Problem solving though group work	-	-	-	146.3±19.06	-	-	-	-	-	77.49±13.28	
19	RCT	102 (E:52, C:50)	E: VR simulation experiences	-	-	5.94±1.46	7.4±1.14	-	-	-	-	2.94±0.39	7.4±1.14	Moderate 10/13
			C: Traditional teaching	-	-	4.30 (1.43)	4.78 ±1.16	-	-	-	-	2.72±0.27	4.78±1.16	
29	RCT	42 (E:21, C:21)	E: Web-based serious game	52.14±19.07	81.19±8.5	2.39±0.88	3.99±0.63	3.35±0.55	4.16±0.39	-	-	3.50±1.97	7.91±1.34	Moderate 10/13
			C: Case-based self-directed learning using e-books.	53.33±13.54	71.42±11.95	2.60±0.64	3.46±0.61	3.22±0.42	3.23±0.36	-	-	3.54±1.69	5.84±1.2	
30	RCT	136 (E:68, C:68)	E: Web based learning	20.5±9.8	32.2±10.5	-	-	-	-	10.5±5.2	18.2±4.9	-	-	High 12/13
			C: Traditional Face to face learning	20.6±10.2	22.1±10.2	-	-	-	-	9.9±4.8	11.7±5.2	-	-	
31	RCT	122 (E:63, C:59)	E: Online education and game-based virtual reality phone applications	10.4±2.6	13.2±2.7	-	-	-	-	8.7±2.2	11.2±1.5	-	-	High 13/13
			C: Traditional education	9.5±2.4	12.3±1.1	-	-	-	-	6.1±1.4	9.3±2.5	-	-	
20	RCT	50 (E:25, C:25)	E: VR simulation program	22.79 ± 2.28	23.44 ± 2.28	-	-	5.47±1.47	8.36±1.05	-	-	-	-	High 12/13
C: Routine NICU practice	22.05 ± 3.31	22 ± 3.3	-	-	5.36±1.68	7.46±1.34	-	-	-	-

Footnotes: RCT: Randomized Control Trial; E: Experimental Group; C: Control Group; I-Pretest (Mean & Standard Deviation); II-Posttest (Mean & Standard Deviation); JBI-Joanna Briggs Institute Quality Assessment Tool

**Table 2:** Outlines the characteristics included in the studies

Study Characteristics	Frequency	Percentage	References
*Type of Intervention*			
Virtual Reality Simulation	4	28.57	19,20,22,26
Virtual Reality (Video; Online & game-based phone application)	2	14.29	25,31
Virtual Learning	1	7.14	24
Simulation (Video-based; Zoom learning)	2	14.29	18,21
Web-based game	1	7.14	29
Online Learning	1	7.14	28
AI-Powered Doctor	1	7.14	27
Web‐based (Mobile training; Learning)	2	14.29	23,30
*Conventional Approaches*			
Theoretical lecture and laboratory teaching	8	57.14	18,19,20,21,24,25,30,31
Questionnaire/training booklet/structured guidelines	3	21.43	23,26,28
Self-directed learning using e-books.	1	7.14	29
Offline Low-fidelity simulation	1	7.14	22
Human-controlled Avatar	1	7.14	27
*Study design*			
RCT	14	100.00	18-31
*Setting*			
Government set up	6	42.86	18,21,24,25,27,30
Private set-up/ Deemed Universities	4	28.57	19,22,26,29
Not Specified	4	28.57	20,23,28, 31
*Sample Size*			
10-150	11	78.57	19-23,25-27,29-31
151- 300	2	14.29	24,28
More than 301	1	7.14	18
*Programme duration*			
1-2 Weeks	6	42.86	20,21,23,25,26,27
More than 2 weeks	6	42.86	19,24,28,29,30,31
Not Specified	2	14.29	18,22
*Group Nature*			
Closed group	13	92.86	18-24,26-31
Open Group	1	7.14	25
*No. of facilitators per group*			
1-15 facilitators	4	28.57	18,25,26,28
Not specified	10	71.43	19-24,27,29-31
*The educational program of Group*			
1^st^Year BSc Nursing	2	14.29	22,24
2^nd^Year BSc Nursing	0	0.00	-
3^rd^Year BSc Nursing	4	28.57	23,25,27,29
4^th^Year BSc Nursing	2	14.29	18,31
Under Graduate (Not Specified)	3	21.43	26,28,30
Other Nursing Courses	2	14.29	19,20,21
*Conduction of Group Activities*			
Yes	9	64.29	18,20,21,22,24,25,27,28,31
No	5	35.71	19,23,26,29,30
*Countries Conducted*			
Turkey	1	7.14	21
China	3	21.43	18,22,28
Korea	3	21.43	20,23,29
Spain	1	7.14	24
Japan	1	7.14	25
Taiwan	1	7.14	26
Singapore	1	7.14	27
Jordan	1	7.14	19
Egypt	1	7.14	30
North Cyprus	1	7.14	31
*Research Approach Used*			
Quantitative Research	14	100.00	18-31

### Evaluation of Methodological Quality in Intervention Studies

**Figure 2:**
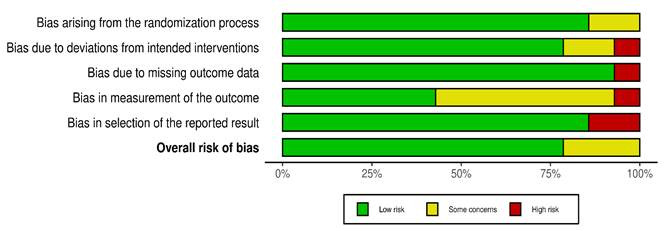
Overall RoB assessment for included RCTs

**Figure 3:**
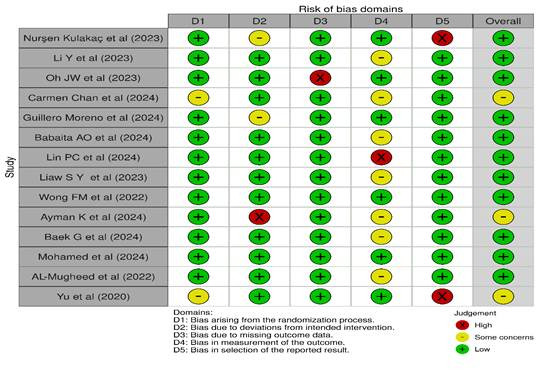
The evaluation of the RoB was conducted for each RCT

Figure 2 summarizes the RoB assessment. Among the 14 studies, 11 consistently show a low RoB throughout most domains. In contrast, three studies raise concerns in particular areas, such as deviations from targeted treatments, insufficient outcome data, and selective outcomes reporting. Figure 3 depicts a complete investigation of the RoB.

### Results of metanalysis

**Figure 4:**
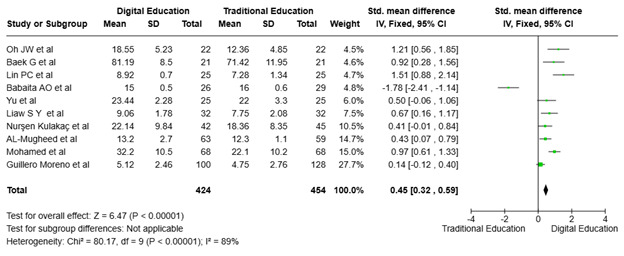
Impact of digital education on nursing students’ cognitive skills (Knowledge)

Effect of digital and traditional interventions on cognitive domain (Knowledge). As shown in Figure 4, 10 RCTs (^20^, ^21^, ^23^–^27^, ^29^–^31^) evaluated the effect of digital technology on nursing students' theoretical knowledge development. The analysis showed significant heterogeneity in included RCTs (p<0.00001; I² = 89%), requiring a random-effects model. The total effect size was calculated to be 0.45 [95% CI: 0.32, 0.59], demonstrating a preference for the experimental group using digital education. The result is statistically significant (p<0.00001) and indicates a moderate positive effect of digital education compared to traditional methods. Studies conducted by Oh et al. (^23^), Baek et al. (^29^), Lin et al. (^26^), and Mohamed et al. (^30^) show positive effects favoring digital education, while others like Babaita et al. (^25^) and Moreno et al. (^24^) show minimal effects, with confidence intervals crossing zero. This variation highlights the influence of study-specific factors on the outcomes.

**Figure 5:**
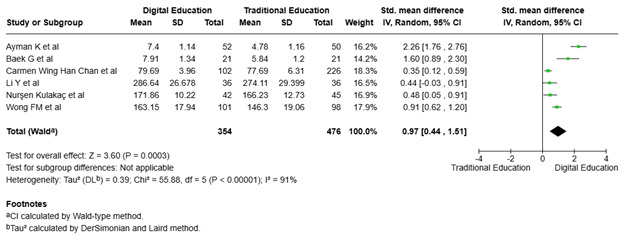
Digital education's impact on nursing students' critical thinking and decision-making

Effect of digital and traditional interventions on critical thinking and clinical decision making. Figure 5 highlights the findings of six studies (^18^, ^19^, ^21^, ^22^, ^28^, ^29^) involving 830 participants randomly assigned to digital education (n=354) and traditional education (n=476). The trials examined the intervention's success in improving critical thinking and clinical decision-making. The meta-analysis found a significant improvement in these domains among nursing students, with a total effect size of 0.97 (95% CI: 0.44, 1.51). Additionally, the heterogeneity was high (I² = 91%), indicating substantial variation between the studies. The total effect was statistically significant (p=0.0003), demonstrating that digital education exceeded traditional techniques.

**Figure 6:**
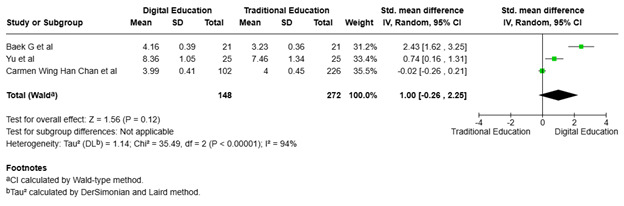
The effect of digital education on problem-solving ability among nursing students

Effect of digital and traditional interventions on problem-solving ability. Figure 6 shows that three studies (^18^, ^20^, ^29^) assessed the impact of digital education on nursing students' problem-solving ability, revealing significant heterogeneity in the involved RCTs (p<0.00001; I² = 94%). As a result, a random-effects model was used in the study, resulting in an overall effect size of 1.00 (95% CI: -0.26, 2.25), indicating a minor preference for digital education. However, the confidence interval contains 0, implying that the result is not statistically significant. The total impact p-value is P = 0.12, indicating no substantial difference between digital and traditional education in terms of the examined result.

**Figure 7:**
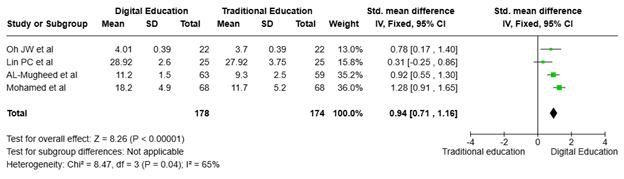
Impact of digital education on nursing students' attitudes

Effect of digital and traditional interventions on affective domain (Attitude). Figure 7 highlights the findings of four studies (^23^, ^26^, ^30^, ^31^) involving 352 participants, randomly assigned to digital education (n = 178) and traditional methods (n = 174). These studies evaluated the intervention's impact on the affective domain, specifically the attitudes of nursing students toward digital education. The standardized mean differences in self-confidence scores before and after the intervention were reported as 0.78 (^23^), 0.25 (^26^), 0.92 (^31^), and 1.28 (^30^), respectively. The meta-analysis found a significant improvement in nursing students' attitudes, with a total effect size of 0.94 (95% CI: 0.71, 1.16). Additionally, the heterogeneity was moderate (I² = 65%), indicating some variation between the studies but not excessively high. The overall effect demonstrated statistical significance (p<0.00001), confirming a notable difference favoring digital education over traditional methods.

**Figure 8:**
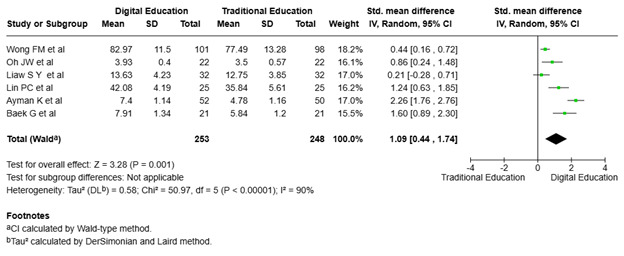
The effect of digital education on improving clinical skills among nursing students

Effect of digital and traditional interventions on the psychomotor domain (Clinical skills improvement). Figure 8 highlights the findings of six studies (^19^, ^23^, ^26^–^29^) involving 501 participants randomly assigned to digital education (n = 253) and traditional education (n = 248). The research investigated how digital simulation affected nursing students' confidence in their clinical performance. The meta-analysis found a total effect size of 1.09 (95% CI: 0.44, 1.74), demonstrating a preference for digital education. The total impact was statistically significant (p<0.001), indicating that digital simulation significantly improves clinical performance and confidence in nursing students compared to traditional education methods. However, heterogeneity was high (I² = 90%, p<0.00001), indicating considerable variability among the studies. Salameh et al. (^19^) and Baek et al. (^29^) found more significant benefits, but Wong et al. (^28^) and Liaw et al. (^27^) observed more moderate effects. This variation implies that study-specific variables, such as intervention design or population characteristics, may have influenced the results.

## Discussion

The primary objective of this systematic review and meta-analysis was to evaluate and analyse key data on the efficacy of using digital instructional tools for nursing students at diverse institutions. According to our findings, this study provides evidence for the beneficial effects of experimental interventions that use digital teaching tools to improve nursing students' cognitive capabilities, including knowledge acquisition, critical thinking, clinical decision-making, and problem-solving abilities. Additionally, it investigated the affective domain, evaluating students' attitudes toward digital educational tools, and the psychomotor domain, investigating the improvement of clinical skills after exposure to digital learning approaches against traditional teaching methods. This detailed review sheds light on the numerous benefits of using digital tools in nursing education.

### The Effect of Digital Education on Nursing Students' Knowledge

A meta-analysis revealed that using digital educational technology in nursing education significantly enhanced cognitive capabilities compared to traditional teaching techniques (*p*<0.05). Simultaneously, the theoretical framework of nursing education is still crucial for preparing nurses to integrate knowledge into practical skills, providing a core component of nursing pedagogy. Nurse educators steadily implemented digital instructional technologies to enhance the impact of theoretical instruction. Chavez *et al.*^32^ and Liu *et al.*^33^ discovered that digital technologies improve student engagement by offering immersive learning experiences akin to real-world clinical settings. 

Furthermore, a qualitative study on the employment of digital advancements in nursing curricula^34^ suggests that combining digital tools with conventional teaching methods allows students to interact with elements in a simulated digital setting, evoking experiences and emotions similar to those encountered in real-world situations. This immersive strategy improves learners' understanding of learned concepts and ability to apply them successfully in clinical situations. According to Kolb's instructional paradigm, nursing students gain essential insights from digital learning experiences that resemble real-life circumstances, leading to more substantial and durable learning outcomes.^35^ According to Chen *et al.*^36^ and Voutilainen *et al.,*^37^ digital education effectively increases information acquisition. Still, it has the most impact when used in conjunction with traditional teaching approaches. Furthermore, using digital technology in nursing education improves student interest and attention to studying while expanding their knowledge base and practical nursing competencies. This integration is a critical step in shifting from knowledge-centered education to competency-driven practice in nursing.

### Digital Education's Impact on Nursing Students' Critical Thinking and Decision-Making

The results demonstrated that digital education interventions significantly improved critical thinking and clinical decision-making skills compared to traditional teaching approaches (*p*<0.05). This indicates how digital teaching tools can effectively bridge the gap between theoretical knowledge and practical application in nursing education. The observed heterogeneity amongst research (I² = 89%) is due to variances in implementation and lack of standardization in digital teaching methodologies. Further inquiry is needed to establish consistent best practices. Similarly, studies have highlighted the benefits of blended and digital learning methodologies for developing critical thinking skills. According to Voutilainen *et al.,*^37^ blended learning systems that combine digital education with traditional teaching methods considerably improve knowledge acquisition and skill development compared to conventional methods alone. Similarly, Saghafi *et al.*^38^ conducted a meta-analysis to highlight the usefulness of digital simulations in improving nursing students' clinical reasoning and critical thinking skills.

Furthermore, the findings of Li *et al*.^39^ highlight the transformational significance of immersive technologies in enhancing critical thinking tendencies in nursing students. Bagheri *et al.*^40^ investigated the effects of network-based learning on nursing students' critical thinking skills. Their findings revealed a statistically significant improvement in critical thinking scores in the intervention group (373.28 ± 18.55) compared to the control group (340.2 ± 10.38, P < 0.001), highlighting the effectiveness of problem-solving-focused digital learning tools. In addition, Sterner *et al.*^41^ reported a significant improvement in critical thinking scores post-education (*p*<0.001) with a large effect size (Cohen’s d = −0.87). In addition, Jans *et al.*^42^ conducted an integrative review that synthesized the evidence highlighting the capacity of virtual reality (VR) to enhance critical thinking, clinical reasoning, and judgment, with students expressing positive perceptions of VR's role in fostering these skills. While digital education technologies and immersive learning environments have demonstrated their effectiveness in nurturing critical thinking and clinical decision-making skills, challenges such as variability in implementation and cognitive load persist. Addressing these problems through properly developed digital and blended learning systems is critical to fully leveraging their promise in nursing education.

### Effect of digital and traditional interventions on the affective domain (Attitude)

The results of this meta-analysis show that digital education is much more effective than traditional approaches in improving nursing students' attitudes, as demonstrated by an SMD of 0.94 (95% CI: 0.71 to 1.16). This finding is consistent with earlier studies emphasizing the value of digital education in developing empathy, emotional engagement, and decision-making skills in nursing practice. This is consistent with a systematic study that investigated how digital interventions improve the emotional abilities of nursing students and registered nurses compared to traditional training methods.^43^ A recent study by Efendi *et al.*^44^ further underscores the efficacy of digital interventions in enhancing nursing staff's affective competencies. The findings align with the outcomes observed in this study, indicating that digital education is a promising tool for fostering emotional engagement, empathy, and trust-building, key components of nursing competence. 

### Effect of digital and traditional education on the psychomotor domain (clinical skills enhancement)

Incorporating digital technology into nursing education has resulted in a considerable improvement in nursing students' psychomotor abilities compared to traditional teaching methods. Liu *et al.*^33^ emphasized that digital education offers an immersive, interactive platform that promotes emotional involvement and comprehension, successfully bridging the gap between theoretical knowledge and practical application. Their meta-analysis revealed a marked improvement in the clinical skills acquirement (SMD = 0.52, 95% CI [0.33, 1.46], *p*<0.001). These findings are congruent with Choi KS,^45^ who demonstrated the benefits of digital-based instructional technology. Similarly, Jefferson *et al.*^46^ showed increased learning retention and self-confidence among nursing students who took high-fidelity simulation courses, highlighting the importance of simulation-based education in improving critical thinking and practical aptitude. Furthermore, Azher *et al.*^47^ found that both headset-based digital education and screen-based virtual simulations provide similar benefits in nursing education. These tools efficiently simulate complex clinical circumstances, allowing students to practice approaches that would be impractical with actual patients, establishing a connection between clinical experience and classroom theory. This promotes a more seamless transition from student to practitioner while overcoming the inherent constraints of traditional training techniques.

Future efforts should focus on improving digital education platforms to promote seamless skills transfer from digital environments to real-world clinical settings. Allowing nursing students to interact with virtual patients enhances their clinical expertise and enables them to deal with tough challenges effectively in real-world situations. Furthermore, using digital education and simulation technologies can speed up the development of both technical and emotional competencies, increasing learning efficiency and overall instructional efficacy in nursing education.

### Strengths and limitations of the study

The findings of this study are derived from a rigorous randomized controlled trial, which provides a more substantial evidentiary basis compared to cohort studies. This high-quality evidence corresponds to the tenets of evidence-based medicine and establishes a concrete framework for the future integration of digital educational technologies into nursing education. Despite its contributions, this meta-analysis encountered some limitations. The variability in study results led to challenges in making direct comparisons, requiring cautious interpretation of the findings and limiting the ability to generalize the effects on nursing education. 

## Conclusion

This comprehensive review and meta-analysis found that digital educational tools can considerably improve nursing students' knowledge, attitudes toward digital learning, clinical competence, critical thinking, and clinical decision-making skills in patient care. However, no significant advantage was observed in improving their problem-solving abilities. The findings advocate for nursing educators to realign their teaching strategies, emphasizing the importance of digital education and actively integrating advanced technological tools to drive educational progress. This review recommends that nursing educators incorporate digital technology into blended classroom teaching. This approach combines the emotional human touch of traditional education with the flexibility of digital platforms, enabling students to learn at their own pace. Generation Z is highly comfortable with digital learning environments, so this method aligns well with their learning preferences.

Author contribution statement. Mathivanan JR and Devi S developed the systematic review and search strategy, carried out the database searches, took part in selecting relevant articles, and Devi S handled the quality assessment of the studies. Both authors contributed to drafting the manuscript.
